# Incidence, lethality, and post-stroke functional status in different Brazilian macro-regions: The SAMBA study (analysis of stroke in multiple Brazilian areas)

**DOI:** 10.3389/fneur.2022.966785

**Published:** 2022-09-15

**Authors:** Emily dos Santos, Giulia M. Wollmann, Vivian Nagel, Herminia M. S. Ponte, Luis E. T. A. Furtado, Rui K. V. Martins-Filho, Gustavo Weiss, Sheila C. O. Martins, Leslie E. Ferreira, Paulo H. C. de França, Norberto L. Cabral

**Affiliations:** ^1^Postgraduate Program on Health and Environment, University of the Region of Joinville–Univille, Joinville, Brazil; ^2^Department of Medicine, University of the Region of Joinville–Univille, Joinville, Brazil; ^3^Joinville Stroke Registry, Hospital Municipal São José, Joinville, Brazil; ^4^Department of Nursing, Inta University Center–UNINTA, Sobral, Brazil; ^5^Department of Clinical Medicine, Federal University of Ceará, Sobral, Brazil; ^6^Hospital das Clínicas de Ribeirão Preto, University of São Paulo–USP, Ribeirão Preto, Brazil; ^7^Hospital de Clínicas de Porto Alegre, Federal University of Rio Grande do Sul, Porto Alegre, Brazil; ^8^Hospital Moinhos de Vento, Porto Alegre, Brazil

**Keywords:** stroke, epidemiology, incidence, lethality, functional status

## Abstract

**Background:**

Stroke is the second leading cause of death in Brazil. The social and financial burden of stroke is remarkable; however, the epidemiological profile remains poorly understood.

**Objective:**

The aim of this study was to report the incidence, lethality, and functional status at 30 and 90 days post-stroke in the cities of different Brazilian macro-regions.

**Methods:**

This is an observational, prospective, and population-based study, led in Canoas (South), Joinville (South, reference center), Sertãozinho (Southeast), and Sobral (Northeast) in Brazil. It was developed according to the three-step criteria recommended by the World Health Organization to conduct population-based studies on stroke. Using different sources, all hospitalized and ambulatory patients with stroke were identified and the same criteria were kept in all cities. All first events were included, regardless of sex, age, or type of stroke. Demographic and risk factor data were collected, followed by biochemical, electrocardiographic, and radiological test results. Functional status and lethality were obtained using the mRankin scale through telephonic interview (validated Brazilian version).

**Results:**

In 1 year, 932 stroke cases were registered (784 ischemic stroke, 105 hemorrhagic stroke, and 43 subarachnoid hemorrhage). The incidence rates per 100,000 inhabitants, adjusted for the world population, were 63 in Canoas, 106 in Joinville, 72 in Sertãozinho, and 96 in Sobral. The majority (70.8%) were followed for 90 days. Kaplan–Meier curves showed that 90-day survival was different among cities. Sobral, which has the lowest socioeconomic indexes, revealed the worst results in terms of lethality and functional status.

**Conclusion:**

This study expands the knowledge of stroke epidemiology in Brazil, a middle-income country with enormous socioeconomic and cultural diversity. The discrepancy observed regarding the impact of stroke in patients from Joinville and Sobral highlights the need to improve the strategic allocation of resources to meet the health priorities in each location.

## Introduction

Since 2005, the prevalence of stroke has increased considerably, and more than 104 million people live with its consequences worldwide (1). The social and financial burden of stroke is remarkable (2). Acute stroke interventions, such as stroke units and reperfusion therapy, have the potential to improve outcomes. In addition, primary prevention is essential to reduce its incidence, prevalence, lethality, and life years lost due to disability (DALYs) (3).

In 2018, the first Latin American Stroke Ministerial Meeting gathered the health ministries from 13 different countries in Brazil to discuss and identify ways to cooperate on reducing stroke charges in the region (4). Meta-regression data showed that the incidence of stroke in Latin America decreased by 25% from 1990 to 2017 (4).

One-third of the Latin America's population is in Brazil, where the stroke incidence per 100,000 inhabitants was reduced from 146 (95% CI: 130–163) in 1990 to 136 (95% CI: 122–153) in 2019 (5). However, the improvements were not enough to eliminate health inequities, and the number of stroke cases usually remains higher in the North and Northeast than in the South and Southeast regions (6). As in other middle-income countries, stroke care follows the social disparities that divide Brazil into two different “countries”: the wealthier part, which shares the same profile as developed countries and can afford high-quality stroke care resources, and the majority of the population, who have various limitations in accessing stroke prevention, acute treatment, and rehabilitation (7). The Brazilian Stroke Society points out that population-based studies are needed in all major regions of the country, from the Amazon to the South, in order to provide more reliable information about the impact of stroke care policies (7).

Given all these worries and social inequality, yet significant in Brazil, this study aimed to report the post-stroke incidence, lethality, and functional status in different macro-regions, comparing population data from Joinville (the reference stroke center in Brazil) vs. other studied cities.

## Methods

### Study framing and planning

The Health Ministry (HM) ordered the Brazilian Stroke Society to expand the knowledge on stroke incidence and prognosis outside the reference centers, so that the influence of socioeconomic diversity could be considered. Joinville Stroke Registry (JOINVASC) was defined as the coordinator of the study due to its population-based databank in progress since 1995, supported by municipal law since 2013 (8, 9). Initially, the HM defined that the study should represent each of the five macro-regions of the country. The cities included in the study had as prerequisites the availability of more than 100,000 inhabitants and the availability of 24-hour access to a cranial tomography (CT), laboratory for biochemical analysis, electrocardiogram (ECG), and conventional radiology. The chosen cities were Canoas (South), Joinville (South), Sertãozinho (Southeast), Sobral (Northeast), and Campo Grande (Midwest). None of the northern cities met the initial inclusion criteria.

A JOINVASC team was sent to the selected cities to lead meetings with hospital managers and municipal health departments. Face-to-face meetings were held to train the local teams according to the three-step criteria recommended by the World Health Organization (WHO) to conduct population-based studies on stroke (10).

### Study design

This is an observational, population-based, prospective, and multicenter study. The events were registered consecutively in Sertãozinho (April 2015 to March 2016), Sobral (January to December 2015), Campo Grande (June 2015 to May 2016), Canoas (January to December 2016), and Joinville (January to December 2016), covering all seasons. The detailed methods regarding population-based studies on stroke have been previously reported (8, 11). Stroke was defined according to WHO criteria (12). The stroke investigation routine followed the Brazilian Society of Cerebrovascular Diseases guidelines (13) and adapted to each hospital's resources and reality. Supplementary Table 1 shows the demographics and aging-related indexes of attending cities.

Using different sources, all hospitalized and ambulatory patients with stroke were identified (14). The same criteria were followed in all cities. To evaluate the hospitalized cases daily, the local research nurse registered all stroke cases with the diagnosis made by a neurologist and confirmed by a head CT. By using the electronic registries of diagnoses listed according to the tenth International Classification of Diseases (CID-10) revision, all death certificates that had any reference to the CID-10 codes related to stroke (161–169), or death for unknown cause (R99), were analyzed every month. The non-identified death causes were investigated through medical records; in this sense, we excluded all patients with sudden death at home, no CT confirming the diagnosis, no medical history or incompatible with the one extracted by the nurse, and patients codified with R99 who were still inconclusive after the medical record revision. To identify mild stroke cases among those who did not seek hospital assistance, general clinicians, cardiologists, neurologists, and neurosurgeons were personally invited to notify the study team of these cases. The local health departments sent a formal declaration of adhesion to highlight the study's importance to the assistance network and the public interest in the data by the HM. It did not perform a direct search of patients at stroke risk, like those with carotid and coronary investigation for diagnostic and therapeutic purposes.

All patients diagnosed with any type of ischemic stroke (IS), hemorrhagic stroke (HS), or subarachnoid hemorrhage (SAH) and residents in one of the studied cities, regardless of age, were included. Permanent residents who had an event outside the city limits were confirmed retrospectively and included in the study. Stroke cases who died within the first 24 h of symptom onset, without having a brain scan confirming the event, were excluded. Additionally, there were excluded patients with subdural and epidural hemorrhages, whether traumatic or not; intracerebral hemorrhages secondary to rupture of arteriovenous malformation; hemorrhages secondary to bleeding by use of oral anticoagulants or by tumor bleeding. Patients without brain images were classified as indeterminate cases and coded as IS (15).

The at-risk population data during 2015 and 2016 were extracted from the Department of Informatics of the Unified Health System (DATASUS/MS) (16) of HM and the municipal health department of each city. Permanent residents were defined as those who lived for at least 12 months in the city before study enrollment.

### Diagnostic and evaluation criteria

Demographic and risk factor data were collected, followed by biochemical, electrocardiographic, and radiological test results. The research nurses registered the cardiovascular risk factors during face-to-face meetings with patients or family members. The neurologist assistant informed the nurse about the clinical stroke syndrome according to the Oxfordshire Community Stroke Project (OCSP) classification (17). The assistant also informed about the corresponding pathophysiological diagnosis according to “Trial of Org 10172 in Acute Stroke Treatment” (TOAST) criteria, used to classify ischemic stroke into five subtypes, namely, atherosclerosis of large arteries (atherothrombotic), cardioembolism (cardioembolic), small vessel occlusion (lacunar), stroke of other determined etiology, and stroke of undetermined etiology (18). The clinical severity of the events was measured by the “National Institutes of Health Stroke Scale” (NIHSS) at hospital admission, stratified into minor (0–3), moderate (4–10), and severe (>10) (19). All criteria were maintained throughout the study.

The education level of patients was stratified on complete school years and skin color by self-definition, according to categories–black, brown, indigenous, white, and yellow – of the Brazilian Institute of Geography and Statistics (IBGE). The economic strata were based on the Brazilian Association of Research Companies classification (20).

The Modified Rankin Scale (mRS) was used to evaluate post-stroke functional dependency and lethality, stratifying patients into three categories: independent (0–2), dependent (3–5), and dead (6) (21). Each nurse, in their respective city, reported the functional status of patients during the first month. After 3 months of the event, a JOINVASC nurse contacted all patients (or family members), using a validated Brazilian version of mRS, to evaluate the functional status through telephonic interview (20).

### Statistical analysis

Results of descriptive analyses are presented as average and standard deviations for quantitative variables and as frequencies, expressed in percentages, for qualitative variables. To calculate incidence and corresponding confidence intervals (CI) of 95%, the Poisson distribution was used for the number of events. The incidence specified by age and sex was adjusted by the direct method for the Brazilian population and for the WHO standard population. The incidence rates were compared using the epidemiological calculator OpenEpi (7, 8). For comparisons among cities, we applied the chi-square test for qualitative variables and the ANOVA test, with Bonferroni correction, for quantitative variables, with *p* < 0.05 considered significant. To analyze the survival rate, Kaplan–Meier curves were plotted (log-rank test). For all these analyses, the statistical software SPSS version 23.0 was used.

### Ethical aspects

This study was approved by the Brazilian Research Ethics Commission, under opinion 759670, on 29 April 2014. The Ethics Committees from each involved city have also approved it locally. Written informed consent was obtained from all study participants or their legal representatives.

## Results

**Figure 1:**
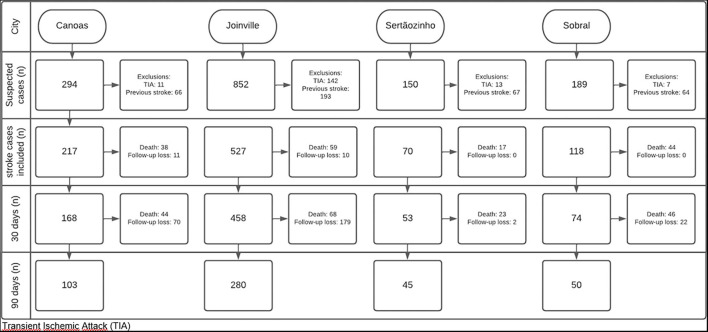
Flowchart of the study follow-up.

A total of 932 new cases of stroke met the inclusion and exclusion criteria of the study from Joinville – reference center (*n* = 527), Canoas (*n* = 217), Sertãozinho (*n* = 70), and Sobral (*n* = 118). The city of Campo Grande (Midwest macro-region) was excluded from the intended analysis as it was not possible to achieve a significant coverage of stroke cases. The 30-day post-event follow-up was carried out in 911 cases. In 90 days, this amount was reduced to 659 cases (Figure 1). Table 1 shows baseline demographic and socioeconomic data of patients, as well as the main risk factors and access to stroke assistance.

**Table 1:** Baseline demographic and socioeconomic data of patients, risk factors, and access to stroke assistance in four Brazilian cities.

	**Canoas (*N* = 217)**	**Joinville (*N* = 527)**	**Sertãozinho (*N* = 70)**	**Sobral (*N* = 118)**	***p***
	***n* (%)**	***n* (%)**	***n* (%)**	***n* (%)**	
**Age, y–mean age (SD)**	64.4 (13.4)	65.2 (14.3)	64.8 (15.4)	66.1 (16.5)	0.785
<45	16 (34.7)	43 (8.2)	6 (8.6)	14 (11.9)	0.848
<55	48 (22.1)	116 (22.0)	16 (22.8)	29 (24.6)	0.964
**Men**	109 (50.2)	283 (53.7)	37 (52.8)	60 (50.8)	0.829
Skin color*		
Black	14 (6.5)	14 (2.7)	6 (8.6)	20 (17.1)	<0.001
Brown	32 (14.7)	18 (3.4)	24 (34.3)	64 (54.7)	<0.001
Indigenous	4 (1.8)	0	0	2 (1.7)	0.013
White	165 (76.0)	493 (93.7)	39 (55.7)	31 (26.5)	<0.001
Yellow	2 (0.9)	1 (0.2)	1 (1.4)	0	0.254
**Years of education** ^†^					
<4 or illiterate	111 (51.4)	322 (61.1)	47 (68.1)	90(78,9)	<0.001
4–8	72 (33.3)	77 (14.6)	6 (8.7)	9 (7.9)	<0.001
8–11	29 (13.4)	95 (18.0)	12 (17.4)	13 (11.4)	0.209
>11	4 (1.9)	33 (6.3)	4 (5.8)	2 (1.8)	0.026
**Social class**		
A	2 (0.9)	7 (1.3)	0	0	0.464
B1	4 (1.8)	13 (2.5)	5 (7.1)	4 (3.4)	0.113
B2	59 (27.2)	115 (21.8)	22 (31.4)	12 (10.2)	0.001
C1	63 (29.0)	170 (32.3)	18 (25.7)	13 (11.0)	<0.001
C2	61 (28.1)	129 (24.5)	11 (15.7)	30 (25.4)	0.219
D	27 (12.4)	92 (17.5)	13 (18.6)	55 (46.6)	<0.001
E	1 (0.5)	2 (0.4)	1 (1.4)	4 (3.4)	0.012
**Alcohol use**	35 (16.1)	35 (6.7)	22 (31.4)	16(13.6)	<0.001
**Physical activity**–**Low**	210 (96.8)	483 (91.7)	50 (71.4)	103 (87.3)	<0.001
**Physiological factors**		
High BMI (> 23)^‡^	178 (82.0)	407 (77.2)	47(67.1)	84 (71.2)	<0.001
High fasting glucose (≥ 5.6 mmol/L)^§^	89 (90.8)	470 (91.4)	15 (100)	2 (100)	0.646
High systolic pressure (≥140 mmHg)	175 (85.4)	477 (90.5)	57 (89.1)	88(91.7)	0.461
High total cholesterol (≥6.2 mmol/L)^¶^	31 (21.2)	50 (10.9)	0	0	0.001
Previous atrial fibrillation	1 (0.5)	45 (8.5)	4 (5.7)	0	<0.001
Previous myocardial infarction	14 (6.4)	38 (7.2)	5 (7.1)	3 (2.5)	0.314
**Tobacco smoke**		
Smoking	124 (57.1)	266 (50.5)	34 (48.6)	58 (49.1)	0.324
Secondhand smoke^**^	76 (35.0)	153 (29.0)	35 (50.0)	30 (25.4)	0.001
**Symptom-to-door time (Hours)**	04:48	06:25	05:59	05:14	
**SAMU**	69 (31.9)	291 (55.7)	23 (32.9)	58 (49.2)	<0.001
**Hospital type**					
Public	179 (83.6)	415 (78.9)	0 (0.0)	0	<0.001
Private	0	111 (21.1)	4 (6.9)	0	<0.001
Mixed (Public/Private)	35 (16.4)	0	54 (93.1)	56 (100.0)	<0.001

SD, standard deviation; SAMU, emergency mobile care service; BMI, body mass index. Data unavailable: *Skin color: Joinville (n = 1; 0.2%) and Canoas (n = 1; 0.8%).

† Years of education: Canoas (n = 1; 0.5%) and Sertãozinho (n = 1.4%).

‡ BMI: Canoas (n = 1; 0.5%), Joinville (n = 3; 0.6%), and Sertãozinho (n = 10; 14.1%).

§ Glucose: Canoas (n = 119; 54.8%), Joinville (n = 13; 2.5%), Sertãozinho (n = 55; 78.6%), and Sobral (n = 116; 98.3%).

¶ Cholesterol: Canoas (n = 71; 32.7), Joinville (n = 67; 12.7%), Sertãozinho (n = 70; 100.0%), and Sobral (n = 118; 100.0%).

** Secondhand smoke: Joinville (n = 2; 0.4%). Symptom-to-door time: Canoas (n = 1; 0.4%) and Sertãozinho (n = 4; 5.7%). Hospital type: Canoas (n = 3; 2.2%), Joinville (n = 1; 0.7%), Sertãozinho (n = 70; 51.1%), and Sobral (n = 62; 52.5%).

**Figure 2:**
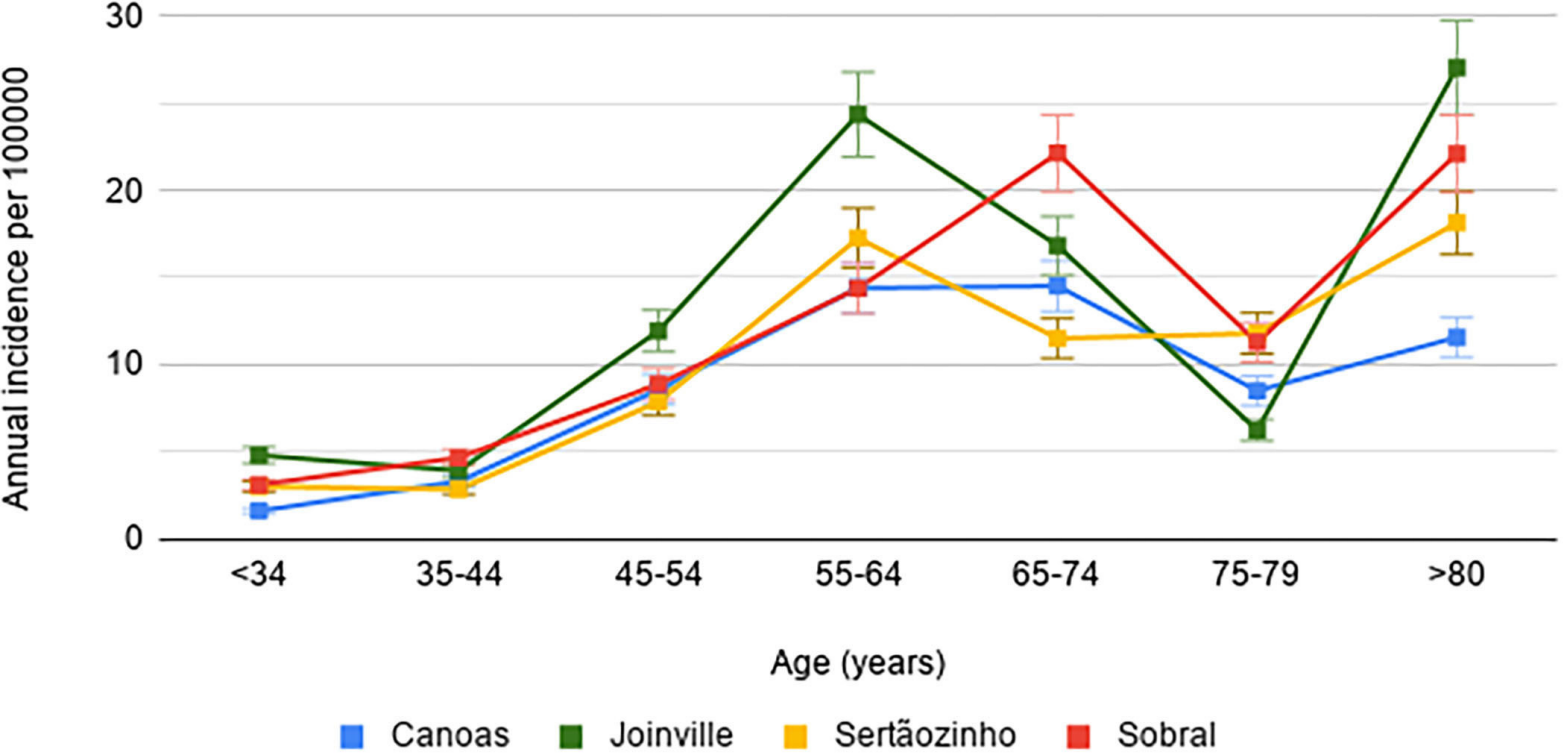
Stroke incidence rates (95% CI) adjusted for the world population in four Brazilian cities.

The obtained stroke incidence rates per 100,000 inhabitants, adjusted for the Brazilian population, were 54.3 (95% CI: 49.3–64.3) in Canoas; 85 (95% CI: 77.9–92.5) in Joinville; 58.7 (95% CI: 49.1–78.2) in Sertãozinho; and 77 (95% CI: 63.7–92.2) in Sobral, with no significant difference between sexes. The stroke incidence stratified by city, age group, and sex, as well as considering adjustment for the world population, is presented in Table 2 and Figure 2. Table 3 shows the relative incidences by age group, comparing the reference center with the other participating cities.

**Table 2:** Stroke incidence rates per 100,000 inhabitants according to age and sex in four Brazilian cities.

**Age strata**	**Canoas**		**Joinville**		**Sertãozinho**		**Sobral**	
**(years)**	**N/N at risk**	**Rate (95% CI)**	**N/N at risk**	**Rate (95% CI)**	**N/N at risk**	**Rate (95% CI)**	**N/N at risk**	**Rate (95% CI)**
Men								
<35	0/95,634	0 (0.1–4.4)	5/168,548	3 (1–7)	0/33,974	0 (0.1–4.4)	4/65,714	6.09 (1.7–15.6)
35–44	4/25,070	16 (4.3–40.9)	10/44,334	22.6 (10.8–41.6)	3/9,419	31.9 (2.6–76.7)	4/13,401	29.9 (8.1–76.4)
45–54	12/21,155	56.7 (29.3-99.1)	40/35,918	111.4 (79.6–151.7)	4/7,588	52,7 (21.4–153.8)	8/9,580	83.5 (36.1–164.5)
55–64	44/17,067	257.8 (187.3–346.1)	79/20,926	377.5 (298.9–470.5)	10/5,366	186.4 (198.8–530.2)	12/5,356	224.1 (115.8–391.4)
65–74	34/8,849	384.2 (266.1–536.9)	80/8,946	894.3 (709.1–1,113)	7/2,585	270.8 (108.9–557.9)	15/2,950	508.5 (284.6–838.6)
75–79	10/2,131	469,3 (193.1–801.7)	31/2,283	1,357.9 (922.6–1,927.4)	3/653	459.4 (94.7–1,342.6)	6/925	648.7 (238–1,411.8)
≥ 80	16/1,858	861.1 (451.9–1,331.6)	33/1,980	1,666.7 (1,147.3–2,340.7)	9/645	1,395.3 (251.7–1,809.1)	9/906	993.4 (454.2–1,885.7)
Total	120/171,764	69.1 (56.9–82.3)	278/282,935	98.3 (87.1–110.6)	36/60,230	59.8 (47.4–90.4)	58/98,832	58.7 (44.6–75.9)
Age-adjusted to Brazil		58.5 (47.6–68.9)		114.6 (101.5–128.9)		55.4 (43.7–83.2)		72.67 (55.2–93.9)
Age-adjusted to World		86.1 (50.5–73.1)		172.8 (109–138.4)		91.6 (45.3–86.3)		105 (58.4–99.5)
Women								
<35	3/93,754	3.2 (0.1–4.4)	11/162,098	3.2 (0.7–9.4)	2/45,639	6.79 (0–2.3)	0/64,336	4.38 (0.5–15.8)
35–44	9/26,272	34.3 (15.7–65)	17/45,367	37.5 (21.8–60)	1/8,902	11.2 (2.7–81.2)	6/14,489	41.4 (15.2–90.1)
45–54	20/23,679	84.5 (51.6–130.4)	33/37,449	88.1 (60.7–123.8)	6/7,689	78 (21.1–151.8)	7/10,705	65.4 (26.3–134.7)
5–64	15/19,810	75.7 (42.4–124.9)	40/23,019	173.8 (124.1–236.6)	11/5,581	197.1 (11.1–157.1)	7/6,536	107.1 (43.1–220.7)
65–74	26/11,817	220 (143.7–322.4)	68/11,248	295.4 (229.4–374.5)	6/3,061	196 (71.9–426.6)	16/4,048	395.3 (225.9–641.9)
75–79	11/3,278	385.6 (233.5–716.6)	32/3,522	284.5 (194.6–401.6)	4/873	458.1 (252.2–1,495.9)	7/1,379	507.6 (204.1–1,045.9)
≥ 80	13/4,191	310.2 (272.9–708)	48/4,006	1,198.2 (883.5–1,588.6)	4/1,119	357.5 (490.7–1,758.9)	17/1,445	1,176.5 (685.3–1,883.6)
Total	97/182,801	53,1(47.5–70.1)	249/28,6709	86.9 (76.4–98.3)	34/72,864	46,7 (33.4–66.8)	60/102,938	58.3 (44.5–75)
Age-adjusted to Brazil		47.8(43–63.5)		77.1 (70.3–90.5)		56,3 (42.2–84.3)		77.1 (58.9–99.3)
Age-adjusted to World		49,8 (42.4–62.7)		90,6 (69.5–89.5)		58,9 (41.9–83.6)		87,9 (58.2–98.2)
All								
<35	3/189,388	1.6 (0.3–4.7)	16/330,646	4.8 (2.7–7.8)	2/66,669	3 (0.4–10.8)	4/130,050	3.1 (0.8–7.9)
35–44	13/51,342	25.3 (12.9–43.3)	27/89,701	30.1 (19.8–43.8)	4/18,321	21.8 (5.9–55.8)	10/27,890	35.9 (17.2–66)
45–54	32/44,834	71.4 (48.8–100.8)	73/73,367	99.5 (78–125.1)	10/15,277	65.5 (31.4–120.5)	15/20,285	73.9 (41.4–121.9)
55–64	59/36,877	160 (121.8–206.4)	119/43,945	270.8 (224.3–324.1)	21/10,947	191.8 (118.7–293.2)	19/11,892	159.8 (96.2–249.5)
65–74	60/20,666	290.3 (221.5–373.7)	148/20,194	336.8 (284.7–395.6)	13/5,646	230.3 (122.6–393.8)	31/6,998	443 (301–628.8)
75–79	21/5,409	388.2 (269.5–638)	63/5,805	312 (239.7–399.2)	7/1,526	458.7 (269.7–1,119.6)	13/2,304	564.2 (300.4–964.8)
≥ 80	29/6,049	479.4 (403–804.7)	81/5,986	1,353.2 (1,074.6–1,681.9)	13/1,764	737 (518.4–1,472.9)	26/2,351	1,105.9 (722.4–1,620.4)
Total	217/354,565	61.25 (55.5–72.4)	527/569,644	92.5 (84.8–100.7)	70/120,150	58.3 (49.8–79.3)	118/20,1770	58.5 (48.4–70.1)
Age-adjusted to Brazil		54.9 (49.3–64.3)		85 (77.9–92.5)		58.7 (49.1–78.2)		77 (63.7–92.2)
Age-adjusted to World		63.4 (54–70.4)		105.8 (85.2–101.2)		71.8 (55.7–88.8)		95.8 (70.4–101.9)

CI, confidence interval.

**Table 3:** Relative incidence rates of stroke by age strata.

**Age, years**	**IRR (95% CI)**		
	**Joinville vs Canoas**	**Joinville vs. Sertãozinho**	**Joinville vs. Sobral**
All <35	3.0 (1.0–13.1)	1.6 (0.4–10.4)	1.6 (0.5–5.5)
All <45	1.5 (0.9–2.9)	1.4 (0.6–3.7)	1.5 (0.6–2.2)
All <55	1.4 (1.0–2.0)^*^	1.5 (0.9–2.6)	1.4 (0.9–2.2)
55–64	1.7 (1.2–2.3)^*^	1.4 (0.9–2.3)	1.7 (1.1–2.8)^*^
65–74	2.5 (1.0–3.4)^*^	3.2 (1.8–5.8)^*^	1.6 (1.1–2.5)^*^
75–79	2.5 (1.6–4.2)^*^	1.8 (0.9–3.9)	1.9 (1.1–3.6)^*^
≥ 80	2.3 (1.6–3.5)^*^	1.5 (0.9–2.6)	1.2 (0.8–1.9)

IRR, incidence rate ratio; CI, confidence interval.

* p <0.05.

The incidence of IS (*n* = 784) per 100,000 inhabitants, adjusted for the Brazilian population, varied significantly (*p* = 0.006) among participant cities, being 45 (95% CI: 37–52) in Canoas; 81 (CI 95%: 74–89) in Joinville; 44 (95% CI: 38–58) in Sertãozinho, and 60 (95% CI: 48–73) in Sobral. The detailed data regarding IS cases are shown in Table 4, Supplementary Table 2, Figure 1. Cases of undetermined etiology were more frequent (41–67%) than other IS subtypes, but showed a significant difference among cities (*p* < 0.001). Considering IS subtypes of defined etiology, according to the TOAST criteria, the cardioembolic subtype was significantly more common (*p* < 0.001) in Joinville, while the lacunar subtype was more common in Sobral (*p* = 0.02). Atherothrombotic stroke cases were not significantly different among participating cities. Regarding the Bamford clinical classification applied to IS cases, significant differences were identified related to the frequencies of all subtypes. Canoas reported a higher frequency of cases classified as partial anterior circulation stroke (PACS) (65.6%, *p* < 0.001), while Sertãozinho had the highest number of total anterior circulation strokes (TACS) (36.5%, *p* = 0.001). Sobral stood out for presenting the highest number of cases of lacunar stroke (LACS) (40.9%, *p* < 0.001) since there were no cases of posterior circulation syndrome (POCS) (*p* = 0.002).

**Table 4:** Ischemic stroke cases and outcomes in one and 3 months after event.

	**Canoas**		**Joinville**		**Sertãozinho**		**Sobral**		***p***
	***N* (%)**	**Mean age (SD)**	***N* (%)**	**Mean age (SD)**	***N* (%)**	**Mean age (SD)**	***N* (%)**	**Mean age (SD)**	
**IS proportion**	180 (82.9)	63.7 (13.3)	460 (87.3)	64.8 (14.3)	52 (74.3)	64.9 (14.3)	92 (78.0)	66.0 (16.6)	0.006
**OCSP**									
LACS	16 (8.9)	64.0 (11.3)	116 (26.0)	64.3 (13.8)	14 (26.9)	62.9 (9.7)	36 (40.9)	66.8 (15.1)	<0.001
PACS	118 (65.6)	64.8 (13.2)	222 (49.7)	64.9 (14.8)	11 (21.2)	63.3 (13.6)	32 (36.4)	59.4 (16.8)	<0.001
TACS	23 (12.8)	64.2 (13.3)	42 (9.4)	62.4 (14.6)	19 (36.5)	65.3 (17.1)	20 (22.7)	74.4 (13.7)	<0.001
POCS	23 (12.8)	57.4 (13.8)	67 (15.0)	65.1 (12.7)	8 (15.4)	69.7 (15.9)	0	-	0.002
TACS vs. non-TACS	0.1	0.1	0.6	0.3	
**TOAST**									
Atherotrombotic	23 (12.8)	66.3 (14.0)	76 (16.5)	65.9 (14.2)	6 (11.5)	65.2 (15.8)	15 (16.5)	69.3 (18.9)	0.557
Lacunar	21 (11.7)	66.8 (10.8)	88 (19.1)	66.4 (13.8)	4 (7.7)	56.7 (6.2)	25 (27.5)	62.8 (16.2)	0.002
Cardioembolic	19 (10.6)	68.0 (11.1)	109 (23.7)	64.0 (14.1)	7 (13.5)	73.3 (12.9)	9 (9.9)	60.8 (15.4)	<0.001
Undetermined	117 (65.0)	61.9 (13.6)	187 (40.7)	64.0 (14.7)	35 (67.3)	64.0 (14.5)	42 (46.2)	67.8 (16.4)	<0.001
**NIHSS**									
Minor (0–3)	90 (50.0)	61.8 (14.0)	234 (50.9)	65.5 (14.4)	12 (23.5)	67.0 (15.8)	21 (23.6)	64.9 (15.5)	<0.001
Moderate (4–10)	39 (21.7)	68.0 (11.4)	145 (31.5)	64.4 (14.0)	15 (29.4)	65.3 (13.4)	25 (28.1)	63.1 (17.6)	0.104
Severe (>10)	51 (28.3)	63.6 (12.7)	81 (17.6)	63.5 (14.6)	24 (47.1)	63.8 (14.8)	43 (48.3)	68.9 (15.5)	<0.001
**mRankin−30 days**									
Independency (0–2)	120 (70.2)	63.6 (13.3)	318 (70.5)	65.2 (14.3)	24 (46.2)	64.2 (14.1)	43 (46.7)	63.1 (18.1)	0.003
Dependency (3–5)	29 (17.0)	64.1 (14.5)	89 (19.7)	65.3 (14.0)	16 (30.8)	65.7 (12.3)	15 (16.3)	70.2 (13.9)	0.117
Lethality (6)	22 (12.9)	65.2 (11.9)	44 (9.8)	60.1 (15.5)	12 (23.1)	65.5 (17.8)	34 (37.0)	68.0 (15.5)	<0.001
**mRankin−90 days**									
Independency (0–2)	71 (59.2)	64.7 (12.6)	192 (64.0)	65.2 (14.5)	24 (47.1)	64.5 (14.1)	29 (39.2)	65.0 (16.4)	0.055
Dependency (3–5)	24 (20.0)	61.3 (13.2)	55 (18.3)	65.8 (15.0)	9 (17.6)	61.7 (12.5)	9 (12.2)	61.3 (21.6)	0.909
Lethality (6)	25 (20.8)	63.2 (12.4)	53 (17.7)	60,7 (15.4)	18 (35.3)	67.3 (15.9)	36 (48.6)	68.9 (15.8)	<0.001

SD, standard deviation; IS, ischemic stroke; OCSP, Oxfordshire Community Stroke Project classification; LACS, lacunar syndrome; PACS, partial anterior circulation syndrome; POCS, posterior circulation syndrome; TACS, total anterior circulation syndrome; TOAST, Trial of ORG 10172 in Acute Stroke Treatment; NIHSS, National Institutes of Health Stroke Scale.

The proportion of functionally independent individuals at 30 days of the IS event, evaluated by mRS, was significantly higher (*p* = 0.003) in Canoas and Joinville. However, this difference in functional status relative to the other cities was not maintained at 90 days after the stroke (*p* = 0.055).

The lethality of IS in 30 and 90 days after event was higher in Sobral (37 and 49%, respectively) and Sertãozinho (23% and 35%), being significantly superior (*p* < 0.001) than observed in the reference center – Joinville (10% and 18%). However, when considering the severity of the stroke, Sobral and Sertãozinho already presented higher proportions (NIHSS > 10: 48.3 and 47.1%, respectively), compared to the cities of Joinville (17.6%) and Canoas (28.3%) (*p* < 0.001).

There were registered 105 HS cases, and the corresponding detailed data are given in Table 5. The incidence per 100,000 inhabitants, adjusted for the Brazilian population, was 7 (95% CI: 3–14) in Sertãozinho; 8 (95% CI: 6–11) in Joinville; 8 (95% CI: 5–11) in Canoas, and 13 (95% CI: 8–20) in Sobral, without a significant difference between sexes. Regarding the functional status of patients affected by HS, there was no significant difference among analyzed cities 30 days after the event. At 90 days of follow-up, Sertãozinho showed a significant proportion of independent patients (62.5%; *p* < 0.001); however, due to the small number of cases (*n* = 5), such a result should be considered sparingly. The cities that presented the highest lethality rates for HS in 30 and 90 days post-event were Canoas (40% and 65%, respectively) and Sobral (50% and 59%), surpassing the reference center – Joinville (16% and 23%), both showing significant differences (*p* = 0.022 and 0.005). More severe cases (NIHSS> 10) were also found mostly (*p* = 0.008) in Canoas (78.1%) and Sobral (70%).

**Table 5:** Hemorrhagic stroke and subarachnoid hemorrhage cases and outcomes in 1 and 3 months after event.

	**Canoas**		**Joinville**		**Sertãozinho**		**Sobral**		***p***
	***N* (%)**	**Mean age (SD)**	***N* (%)**	**Mean age (SD)**	***N* (%)**	**Mean age (SD)**	***N* (%)**	**Mean age (SD)**	
**HS proportion**	32 (14.7)	68.0 (13.4)	44 (8.3)	67.1 (12.0)	9 (12.9)	70.0 (21.1)	20 (16.9)	65.3 (17.1)	<0.001
**NIHSS**									
Minor (0–3)	5 (15.6)	61.8 (14.0)	11 (25.0)	65.5 (14.4)	1 (11.1)	67.0 (15.8)	2 (10)	64.9 (15.5)	0.436
Moderate (4–10)	2 (6.3)	68.0 (11.4)	15 (34.1)	64.4 (14.0)	3 (33.3)	65.3 (13.4)	4 (20.0)	63.1 (17.6)	0.032
Severe (>10)	25 (78.1)	63.6 (12.7)	18 (40.9)	63.5 (14.6)	5 (55.6)	63.8 (14.8)	14 (70.0)	68.9 (15.5)	0.008
**Rankin−30 days**		
Independency (0–2)	7 (23.3)	77.1 (9.5)	19 (43.2)	68.5 (13.7)	5 (55.6)	79.0 (17.4)	6 (30.0)	74.3 (7.6)	0.100
Dependency (3–5)	11 (36.7)	65.0 (18.8)	18 (40.9)	67.3 (11.4)	2 (22.2)	59.0 (38.2)	4 (20.0)	59.2 (12.1)	0.766
Lethality (6)	12 (40.0)	67.3 (7.2)	7 (15.9)	62.8 (8.6)	2 (22.2)	59.0 (1.4)	10 (50.0)	62.4 (21.3)	0.022
**Rankin 90 days**		
Independency (0–2)	3 (13.0)	84.0 (9.6)	11 (35.5)	62.1 (14.8)	5 (62.5)	73.6 (26.8)	2 (11.8)	76.5 (0.7)	<0.001
Dependency (3–5)	5 (21.7)	60.0 (25.8)	13 (41.9)	70.0 (11.4)	1 (12.5)	86.0 (0.0)	5 (29.4)	63.6 (13.1)	0.040
Lethality (6)	15 (65.2)	69.0 (7.6)	7 (22.6)	62.8 (8.6)	2 (25.0)	59.0 (1.4)	10 (58.8)	62.4 (21.3)	0.005
									
**SAH proportion**	5 (2.3)	68.4 (15.7)	23 (4.4)	68.7 (17.4)	9 (12.9)	58.9 (15.3)	6 (5.1)	69.5 (15.6)	0.004
**NIHSS**									
Minor (0–3)	1 (20.0)	79.0 (0.0)	7 (30.4)	69.7 (17.5)	3 (33.3)	60.7 (25.0)	3 (50.0)	60.0 (15.4)	0.743
Moderate (4–10)	0 (0.0)	0 (0.0)	4 (17.4)	60.7 (12.3)	2 (22.2)	64.5 (0.7)	2 (33.3)	84.0 (7.1)	0.551
Severe (>10)	4 (80.0)	65.7 (16.8)	12 (52.2)	70.7 (19.2)	4 (44.5)	54.7 (12.7)	1 (16.7)	69.0 (0.0)	0.204
**Rankin−30 days**									
Independency (0–2)	1 (20.0)	79.0 (0.0)	10 (45.5)	63.3 (20.5)	5 (55.6)	62.0 (17.8)	5 (83.3)	69.6 (17.4)	0.190
Dependency (3–5)	0 (0.0)	0 (0.0)	4 (18.2)	77.0 (2.9)	1 (11.1)	36.0 (0.0)	1 (16.7)	69.0 (0.0)	0.751
Lethality (6)	4 (80.0)	65.7 (16.8)	8 (36.4)	72.0 (17.6)	3 (33.3)	61.3 (3.1)	0 (0.0)	0 (0.0)	0.054
**Rankin−90 days**									
Independency (0–2)	0 (0.0)	0 (0.0)	7 (41.2)	61.6 (21.7)	5 (55.6)	62.0 (17.8)	4 (80.0)	71.0 (19.8)	0.101
Dependency (3–5)	0 (0.0)	0 (0.0)	2 (11.8)	73.5 (10.6)	1 (11.1)	36.0 (0.0)	1 (20.0)	69.0 (0.0)	0.830
Lethality (6)	4 (100.0)	65.7 (16.8)	8 (47.1)	72.0 (17.6)	3 (33.3)	61.3 (3.1)	0 (0.0)	0 (0.0)	0.023

SD, standard deviation; HS, hemorrhagic stroke; NIHSS, National Institutes of Health Stroke Scale; SAH, subarachnoid hemorrhage.

A total of 43 cases of SAH were registered in the four cities, with Sertãozinho standing out, where 13% of events corresponded to this type of stroke (*p* = 0.004). It has not been found that there are significant differences regarding sex, functional status, and lethality in 30 days for SAH. More details on SAH and HS are given in Table 5.

**Figure 3:**
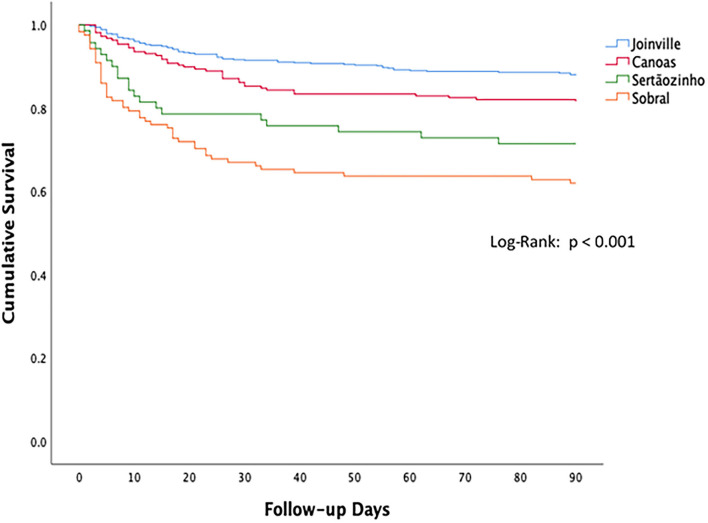
Kaplan–Meier curve showing 90-day cumulative survival after stroke in four Brazilian cities.

The Kaplan–Meier curve (Figure 3) shows the cumulative 90-day survival of patients of all types of stroke, clarifying the significant differences among studied cities (Log-rank *p* < 0.001). The result reinforces that Joinville, the reference center, has the best survival rate, while Sobral, the representative of the Northeast macro-region, shows the worst epidemiological situation.

## Discussion

For the first time, an observational study was able to analyze the incidence and lethality of stroke in medium-sized cities in three Brazilian macro-regions, prospectively and simultaneously, in addition to follow-up of the patients for 90 days to evaluate their post-event functional status. The rate of loss of patient follow-ups was similar to other studies (22).

The demographic and socioeconomic characteristics of the population studied are in line with the findings described in several international studies, in which hospitals in low- and middle-income countries treated patients diagnosed with stroke who were, on average, younger and less educated when compared with high-income countries (23). A trend toward stabilization in the general population or an increase in stroke incidence rates in middle-aged people has been observed more recently in European countries, the United States, Brazil, and China (24).

In this study, a significant variation was observed in the proportion of stroke types among the participating cities, with Joinville having the highest case proportion related to IS (87.7%, *p* = 0.006); Sobral for HS (16.9%, *p* = 0.011); and Sertãozinho for SAH (12.9%, *p* = 0.004), as well as there were significant variations on IS subtypes. Béjot et al. (2016) analyzed contemporary European population-based records and identified huge discrepancies regarding the distribution of stroke types (IS: 55–90%, HS: 10–25%, and SAH: 0.5–5%), and also among the ischemic subtypes (25). Those differences can occur due to ethnic/racial background, variations in the prevalence of cardiovascular risk factors, and socioeconomic and environmental status (26).

Incidence rates per 100,000 inhabitants, adjusted for the world population, found in the cities of Canoas (63), Joinville (106), Sertãozinho (72), and Sobral (96) are in line with the rates described for low- and medium-income countries (60–93), as pointed out in the publication of the “Global Burden of Disease Study 2017” (27).

In China, a North-South gradient was identified, demonstrating a wide variation in stroke within the same country. According to the authors, their results may be related to the differences in socioeconomic conditions, knowledge of the population about the disease, and quality of primary prevention across regions (28). In Brazil, the demographic and epidemiological changes that occurred in the past 50 years have not been experienced uniformly across states, resulting in subnational health disparities and corresponding burdens on health systems (6). In general, there was a decline in transmitted diseases due to the improvements in the public health system in Brazil – SUS, financed by the government and active in prevention and health care. On the contrary, there is an increase in the burden of non-transmittable diseases, in addition to the growth of the elderly population (25, 29).

Previously, there was a little variation in functional status from 3 months to 10 years after stroke (30). In Brazil, there are little comprehensive data on functional status post-stroke (31). The proportion observed in this study of dependent patients (mRS> 2) in 90 days (ischemic: 17.8%, hemorrhagic: 22.9%) was lower than that found in studies carried out in Sweden (ischemic: 33.9%, hemorrhagic: 30.3%) and Iran (ischemic: 28.4%, hemorrhagic: 33.3%) (32, 33). It is well-known today that there is a major impact on the life quality of patients who have survived stroke due to the resulting disabilities, in addition to the emotional and economic factors imposed on them and their families, which means that functional dependence also carries a significant financial burden on health systems (34).

In Brazil, stroke has been the main cause of death for more than 30 years. Although mortality has decreased, it is still occupying the second position, with more than 100,000 deaths per year (35, 36). In this study, the most severe cases were observed in Sobral, in the Northeast macro-region, which also has the highest lethality rate. Factors such as geographical location, race/ethnicity, and the interactions between these various factors, need to be considered (37). Among the studied cities, patients from Sobral presented lower educational levels and the worst socioeconomic conditions, reflected in its lower Human Development Index (HDI). A range of evidence suggests that socioeconomic deprivation is not only associated with the occurrence of stroke and its risk factors but also increases the severity, mortality, and incidence of the event at younger ages (38–40). The higher age-standardized burden of stroke may also be related to poorer acute healthcare for stroke (41).

Stroke care in low- and middle-income countries is patchy, fragmented, and often results in poor patient outcomes (42). Individuals presenting with acute stroke may be severely affected by healthcare coverage, including the availability of ambulance services, stroke units, reperfusion therapy, or rehabilitation in different health settings (3). Despite the advances in the treatment of stroke in recent years, access to diagnosis and treatment remains heterogeneous in Brazil (7). The strategic allocation of financial resources for healthcare, according to disease priorities in each state or macro-region, remains a great challenge (6). Joinville, a reference center in stroke assistance that has a public hospital with an acute and integral stroke unit (U-stroke), despite having a higher incidence when compared to the other cities in the study, had patients with a better degree of functional independence and a lower lethality rate. The interstroke study showed that patient admission to a hospital with a U-stroke is associated with increased chances of survival as well as survival without severe disability. This suggests that stroke units indeed can provide benefits in low- and middle-income countries as observed in high-income countries (23). Improving stroke services is critical for reducing the global stroke burden (43).

The study strengths are related to the prospective capture of all cases, following the three-step criteria proposed by WHO for population-based epidemiological studies on stroke (10). The studied cities belong to three different macro-regions of a middle-income country with large territorial extensions. Furthermore, the study included cities located outside of the stroke reference centers in the country, allowing a better recognition of the impact of the disease in locations with scarce epidemiological data.

On the contrary, the main weakness of this study is related to the absence of equivalent data from the North and Midwest regions, thus not reflecting a comprehensive picture of the country. In addition, difficulties encountered in one or more participating cities may have impacted the results, such as data from private services; lack of complementary diagnostic tests, essential for the research purpose; short hospital stay, due to overcrowding; and lack of specialized stroke treatment units. Another example of difficulty was the temporary unavailability of CT, resulting in high rates of undetermined subtype of IS, due to incomplete investigation. Moreover, it can be assumed that there may have been underreported mild cases seen outside hospitals. Comparisons among cities regarding time to needle were not possible, despite its importance and possible influence on the results presented, since such data were not collected in all participating centers. It is also important to highlight that we had a significant loss of patient follow-up (approximately 30% after 90 days of the event), due to difficulties faced by the teams in each city. Unfortunately, these facts reflect the weaknesses of the Brazilian health system itself.

It is concluded that the observed differences in the incidence and impact of stroke, among cities in different Brazilian macro-regions, are alarming, with particular prominence for the representative city of the Northeast region, where lethality was significantly higher when compared to the stroke reference center located in the South region. This epidemiological reality reflects the need for a better allocation of resources and more effective strategies for healthcare. Therefore, future studies focused on the identification and measurement of differences in access to the diagnosis and treatment of stroke are necessary to discover and possibly remedy the weak points of health assistance in the most deprived Brazilian regions.

## Data availability statement

Primary data will be made available upon reasonable request.

## Ethics statement

This study was approved by the Brazilian Research Ethics Commission, under opinion 759670, from April 29th, 2014. The local ethics committees from each of the participating cities also approved the study. The cities involved were Campo Grande - Federal University of Mato Grosso do Sul, Canoas - Hospital de Clínicas de Porto Alegre, Joinville - University of the Joinville Region, Sertãzinho - Hospital das Clínicas, Faculty of Medicine, University of São Paulo and Sobral - Vale do Acaraú State University. Written informed consent was obtained from all study participants or their legal representatives.

## Author contributions

NC, VN, PF, and LFe contributed to study design. VN, GWo, HP, LFu, RM-F, and GWe contributed to data collection. ES contributed to data analysis, data interpretation, and writing. NC, PF, LFe, and SM contributed to data interpretation and critical revision of the manuscript. All authors contributed to the article and approved the submitted version.

## Funding

This study was made possible with resources from the National Council for Scientific and Technological Development (CNPq Process: 402396/2013-8), the Brazilian Stroke Network (Rede Brasil AVC), and the Foundation for Research and Innovation Support of the State of Santa Catarina (FAPESC) which awarded a scholarship to the researcher ES.

## Conflict of interest

The authors declare that the research was conducted in the absence of any commercial or financial relationships that could be construed as a potential conflict of interest. The reviewer CS declared a shared affiliation with the author RM-F to the handling editor at the time of review.

## Publisher's note

All claims expressed in this article are solely those of the authors and do not necessarily represent those of their affiliated organizations, or those of the publisher, the editors and the reviewers. Any product that may be evaluated in this article, or claim that may be made by its manufacturer, is not guaranteed or endorsed by the publisher.
