# Supplementary material for: Incidence, lethality, and post-stroke functional status in different Brazilian macro-regions: The SAMBA study (analysis of stroke in multiple Brazilian areas)
Source: Front Neurol. 2022 Sep 15;13:966785. doi: 10.3389/fneur.2022.966785 (PMC9520622; doi:10.3389/fneur.2022.966785)
Supplement: Supplementary file 1 [file Data_Sheet_1.docx]

Supplementary Material

**Characterization of the participating cities regarding the structures directly involved in the assistance to stroke cases**

The minimum requirements for the definition of participating cities were the availability of 24-hour access to cranial tomography (CT), biochemical analysis laboratory, electrocardiogram (ECG), and conventional radiology.

Canoas (state of Rio Grande do Sul): At the time of the study, Canoas had 323,827 inhabitants, with an HDI of 0.750 (551st place in Brazil), 818 hospital beds (637 and 181 in the public and private sectors, respectively), and 34 Basic Health Units. Characterization of hospitals with stroke care in Canoas (n=3):

- University Hospital: With extensive facilities, the University Hospital is linked to the campus of the Lutheran University of Brazil (ULBRA), where students in the health area put their learning into practice. With 95% of its care focused on the Unified Health System (SUS), it had services such as CT, magnetic resonance imaging, its own surgical center, among others.

- Emergency Room Hospital: Hospital of the Brazil Stroke Network, focuses on assistance to urgent and emergency cases, and its patients are sent to University Hospital Nossa Senhora das Graças Hospital after being stabilized. It had 110 beds dedicated to the SUS, 10 of which are exclusive to the adult ICU. Additionally, it had a neurology team available 24 hours and performed thrombolysis.

- Nossa Senhora das Graças Hospital: Hospital of the Brazil Stroke Network, which receives patients from the public and private service, with 20 exclusive beds for the adult ICU and a variety of equipment, such as CT. In addition, it had 8 operating rooms and 22 beds for recovery.

Joinville (state of Santa Catarina): population of 515,288 inhabitants and an HDI of 0.809 (21st position in Brazil). In 2016, it had 1114 hospital beds (769 and 345 in the public and private sectors, respectively) and 55 Basic Health Units. City selected as a reference center, being the headquarters of the Joinville Stroke Registry (JOINVASC), which began its activities in 2005. On June 12, 2013, Municipal Law 7448 was enacted, which institutionalized the database within the scope of the municipal public power. Participating hospitals in Joinville (n=5):

-  São José Municipal Hospital: Municipal public hospital, reference in neurology, receiving patients by spontaneous demand or referred from Basic Health Units and emergency service. It had an acute and integral stroke unit, authorized by Ordinance GM/MS nº.665, a neurology team on duty 24 hours a day, 7 days a week, able to perform intravenous thrombolysis, in addition to a vascular neurology team and hemodynamics service with neuroradiologists on call.

- Hans Dieter Schmidt Regional Hospital: It had an External Emergency, Surgical Center, ICU, Day Hospital, in addition to inpatient units. Additionally, it had the clinical specialty of neurology.

- Bethesda Hospital: Philanthropic institution that acts as a back-up hospital, where discharged patients are referred from public hospitals, with only need for rehabilitation or biochemical control.

- Dona Helena Hospital: Private hospital, with 24-hour emergency care, surgical center, laboratory and ICU. It had a neurology service focused on outpatient and hospital care, with tests and procedures specific to the specialty and exams in the area of neurophysiology.

- Unimed Hospital Center: Private hospital, whose structure had 24-hour emergency care, diagnostic imaging center, laboratory, surgical center and ICU.

Sertãozinho (state of São Paulo): 110,074 inhabitants, with an HDI of 0.761 (350th position in Brazil). There were, in 2015, 157 hospital beds (64 and 93 in the public and private sectors, respectively), and 9 Basic Health Units. Participating hospitals in Sertãozinho (n=2):

- Santa Casa de Misericórdia (Holy House of Mercy): It had a reference adult ICU for the care of stroke cases, with neurosurgery and neurology teams.

- Netto Campelo Hospital: It is the only private hospital in the region, with 10 ICU beds and 10 for specialized care. It is a reference in general practice and oncology services, with an advanced center.

Sobral (state of Ceará): It had a population of 188,233 inhabitants and an HDI of 0.714 (1486th position in Brazil). The city had 847 hospital beds available (759 and 88 in the public and private sectors, respectively) and 30 Basic Health Units in 2015. Participating hospitals in Sobral (n=3):

- Santa Casa de Sobral Hospital: Reference philanthropic hospital in the region, receiving demand from patients from all over the state of Ceará to consult at its neurosurgical center.

- Northern Regional Hospital: It was the largest hospital in the interior of the northeast region, receiving a demand of 1.6 million people. It had an urgency and emergency structure available 24 hours a day, covering medium and high complexity cases. In addition, it had a center specializing in neurosurgery and neuroradiology.

- Unimed Hospital: Private hospital with the aim of assisting clients of its health plan. It had a neurology and CT team available 24 hours a day, while high-complexity cases were referred to other hospital providers.

**Supplementary table 1** - City population, area, human development index and respective state epidemiological in 2015.

| City | Population | Area (Km^2^) | HDI  (Brazilian position) | State/Region | State life expectancy (years) (95% CI) | State HALE  (years) (95% CI) | State age-standardised DALY rate per 100 000 (95% CI) |
| --- | --- | --- | --- | --- | --- | --- | --- |
| Canoas | 341,342 | 132 | 0.75 (551) | Rio Grande do Sul/South | 75.7  (74.7-76.9) | 66  (63.1-68.6) | 27 278.1  (24 115-30 620.1) |
| Joinville | 562,153 | 1,131 | 0.81 (21) | Santa Catarina/South | 76.2  (75.0-77.2) | 66.4  (63.4-69.1) | 26 707.6  (23 436.2-30 318.8) |
| Sertãozinho | 120,150 | 403 | 0.76 (350) | São Paulo/Southeast | 76.1  (75.2-77.0) | 66  (63.1-68.9) | 26 880.8  (23 638.3-30 416.8) |
| Sobral | 201,770 | 2,123 | 0.71 (1486) | Ceará/Northeast | 74.8  (73.6-75.9) | 65.1  (62.1-67.8) | 29 370.8  (25 881.7-33 280·2) |
| Brazil | 207 847,528 | 8,516,000 | 0.75 | - | 75.6  (74.7-75.7) | 65.5  (62.5-68.0) | 28 424.7  (25 411.7-31 646.7) |

HDI: human development index; HALE: healthy life expectancy; CI: confidence Interval; DALY: disability-adjusted life-years.

**Supplementary table 2 -** Incidence of ischemic stroke by TOAST classification.

| Age strata (years) | Canoas | | Joinville | | Sertãozinho | | Sobral | |
| --- | --- | --- | --- | --- | --- | --- | --- | --- |
|  | **n/n at risk** | **Rate (95% CI)** | **n/n at risk** | **Rate (95% CI)** | **n/n at risk** | **Rate (95% CI)** | **n/n at risk** | **Rate (95% CI)** |
|  |  |  |  | **IS Atherothrombotic** |  |  |  |  |
| ≤24 | 0/131249 | 0(0-3.7) | 0/226662 | 0(0-3.7) | 0/44575 | 0(0-3.7) | 0/90831 | 0(0-3.7) |
| 25-34 | 0/58139 | 0(0-3.7) | 0/103984 | 0(0-3.7) | 0/22094 | 0(0-3.7) | 0/39219 | 0(0-3.7) |
| 35-44 | 2/51342 | 1.9(0-10.6) | 1/89701 | 1.1(0-6.1) | 0/18321 | 0(0-3.7) | 1/27890 | 3.6(0.1-20.1) |
| 45-54 | 2/44834 | 4.5(1.4-19.6) | 4/73367 | 5.5(1.5-14.1) | 1/15277 | 6.5(0.2-36.2) | 1/20285 | 4.9(0.1-27.3) |
| 55-64 | 5/36877 | 13.6(4.4-31.7) | 26/43945 | 59.2(38.7-86.7) | 2/10947 | 18.3(2.2-66.1) | 2/11892 | 16.8(2-60.7) |
| 65-74 | 7/20666 | 33.9(16.7-76.3) | 30/20194 | 68.3(46.1-97.5) | 0/5646 | 0(0-0) | 4/6998 | 57.2(15.6-146.5) |
| 75-79 | 2/5409 | 37(0.5-103.1) | 8/5805 | 137.8(59.5-271.5) | 2/1526 | 131.1(15.9-473.6) | 3/2304 | 130.2(26.9-380.5) |
| ≥80 | 5/6049 | 82.7(26.9-193) | 7/5986 | 116.9(47-240.9) | 1/1764 | 56.7(1.4-315.9) | 4/2351 | 170.1(46.3-435.5) |
| All | 18/354565 | 6.5(3-8.1) | 76/569644 | 13.3(10.1-16.6) | 6/120150 | 5(1.8-10.9) | 15/201770 | 7.4(4.1-12.2) |
| Age-adjusted to Brazil | | 5.8(2.6-6.9) |  | 13.1(10-16.4) |  | 9.9(5.6-16.4) |  | 9.95.6-16.4) |
| Age-adjusted to World | | 7.1(2.7-7.2) |  | 15.8(11.1-18.3) |  | 6.7(2.3-13.5) |  | 13.1(6.4-18.9) |
| IS Lacunar | | | | | | | | |
| ≤24 | 0/131249 | 0(0-3.7) | 0/226662 | 0(0-3.7) | 0/44575 | 0(0-3.7) | 0/90831 | 0(0-3.7) |
| 25-34 | 0/58139 | 0(0-3.7) | 0/103984 | 0(0-3.7) | 0/22094 | 0(0-3.7) | 0/39219 | 0(0-3.7) |
| 35-44 | 1/51342 | 2(0.1-11.1) | 3/89701 | 3(0.6-8.8) | 0/18321 | 0(0-3.7) | 1/27890 | 4(0.1-22.3) |
| 45-54 | 5/44834 | 11(3.6-25.7) | 10/73367 | 14(6.7-25.7) | 0/15277 | 0(0-0) | 2/20285 | 10(1.2-36.1) |
| 55-64 | 4/36877 | 11(3-28.2) | 27/43945 | 61(40.2-88.8) | 1/10947 | 9(0.2-50.1) | 6/11892 | 50(18.3-108.8) |
| 65-74 | 7/20666 | 34(13.7-70.1) | 26/20194 | 59(38.5-86.4) | 1/5646 | 18(0.5-100.3) | 7/6998 | 100(40.2-206) |
| 75-79 | 2/5409 | 37(4.5-133.7) | 14/5805 | 241(131.8-404.4) | 0/1526 | 0(0-0) | 3/2304 | 130(26.8-379.9) |
| ≥80 | 2/6049 | 33(4-119.2) | 8/5986 | 134(57.9-264) | 2/1764 | 113(13.7-408.2) | 6/2351 | 255(93.6-555) |
| All | 21/354565 | 6(3.7-9.2) | 88/569644 | 15(12-18.5) | 4/120150 | 3(0.8-7.7) | 25/201770 | 12(7.8-17.7) |
| Age-adjusted to Brazil | | 5.3(3.3-8) |  | 15.9(12.7-19.6) |  | 4(1.1-10.2) |  | 16.9(10.9-24.9) |
| Age-adjusted to World | | 5.9(3.5-8.7) |  | 19.3(14.5-22.2) |  | 5.1(1.1-10.2) |  | 21.4(12.2-27.9) |

TOAST: Trial of ORG 10172 in Acute Stroke Treatment; CI: confidence interval; IS: ischemic stroke.

**Supplementary table 2 -** Incidence of ischemic stroke by TOAST classification (continued).

| Age strata (years) | Canoas | | Joinville | | Sertãozinho | | Sobral | |
| --- | --- | --- | --- | --- | --- | --- | --- | --- |
|  | **n/n at risk** | **Rate (95% CI)** | **n/n at risk** | **Rate (95% CI)** | **n/n at risk** | **Rate (95% CI)** | **n/n at risk** | **Rate (95% CI)** |
|  |  |  |  | **IS Cardioembolic** |  |  |  |  |
| <24 | 0/131249 | 0(0-3.7) | 0/226662 | 0(0-43131) | 0/44575 | 0(0-3.7) | 0/90831 | 0(0-3.7) |
| 25-34 | 0/58139 | 0(0-3.7) | 3/103984 | 2.9(0.6-8.5) | 1/22094 | 4.5(0.1-25.1) | 0/39219 | 0(0-3.7) |
| 35-44 | 1/51342 | 1.9(0-10.6) | 2/89701 | 2.2(0.3-7.9) | 0/18321 | 0(0-0) | 1/27890 | 3.6(0.1-20.1) |
| 45-54 | 1/44834 | 2.2(0.1-12.3) | 4/73367 | 5.5(1.5-14.1) | 1/15277 | 6.5(0.2-36.2) | 3/20285 | 14.8(3.1-43.3) |
| 55-64 | 4/36877 | 10.8(2.9-27.7) | 15/43945 | 34.1(19.1-56.2) | 2/10947 | 18.3(2.2-66.1) | 2/11892 | 16.8(2-60.7) |
| 65-74 | 5/20666 | 24.2(7.9-56.5) | 31/20194 | 70.5(47.9-100.1) | 1/5646 | 17.7(0.4-98.6) | 1/6998 | 14.3(0.4-79.7) |
| 75-79 | 5/5409 | 92.4(30-215.6) | 20/5805 | 344.5(210.4-532.1) | 1/1526 | 65.5(1.7-364.9) | 0/2304 | 0(0-0) |
| >80 | 3/6049 | 49.6(10.2-145) | 34/5986 | 568(393.4-793.7) | 1/1764 | 56.7(1.4-315.9) | 2/2351 | 85.1(10.3-307.4) |
| All | 19/354565 | 5.4(3.3-8.4) | 109/569644 | 19.1(15.7-23) | 7/120150 | 5.8(2.3-12) | 9/201770 | 4.5(2.1-8.5) |
| Age-adjusted to Brazil | | 4.8(2.9-7.5) |  | 21.9(18-26.4) |  | 5.8(2.3-12) |  | 5.8(2.7-11) |
| Age-adjusted to World | | 6(3.3-8.7) |  | 31.9(21.6-31.7) |  | 7(2.6-13.4) |  | 6.9(2.8-11.7) |
|  | |  |  |  |  |  |  |  |
| IS undetermined and other cause | | | | | | | | |
| <24 | 1/131249 | 0,8(0-5.6) | 1/226662 | 0,4(0-6) | 0/44575 | 0(0-3.7) | 0/90831 | 0(0-3.7) |
| 25-34 | 1/58139 | 1.7(0.1-11.1) | 6/103984 | 5.8(2.2-13.1) | 1/22094 | 4.5(0.1-27.9) | 2/39219 | 5(0.6-18.1) |
| 35-44 | 10/51342 | 19.5(5.6-28.8) | 18/89701 | 20(11.9-31.6) | 1/18321 | 5.5(0.1-27.9) | 3/27890 | 11(2.3-32.1) |
| 45-54 | 25/44834 | 55.8(20.6-58.5) | 33/73367 | 45(31-63.2) | 8/15277 | 52.4(7.1-66.6) | 4/20285 | 20(5.4-51.2) |
| 55-64 | 32/36877 | 86.8(68.6-135.7) | 38/43945 | 86(60.9-118) | 7/10947 | 63.9(31.5-143.8) | 7/11892 | 59(23.7-121.6) |
| 65-74 | 27/20666 | 130.6(110.1-224.7) | 50/20194 | 114(84.6-150.3) | 9/5646 | 159.4(28.9-207.7) | 11/6998 | 157(78.4-280.9) |
| 75-79 | 9/5409 | 166.4(142.6-193.2) | 14/5805 | 241(131.8-404.4) | 4/1526 | 262.1(232.2-295.7) | 4/2304 | 174(47.4-445.5) |
| >80 | 12/6049 | 198.4(36.3-215.5) | 27/5986 | 451(297.2-656.2) | 5/1764 | 283.4(251.9-317.9) | 11/2351 | 468(233.6-837.4) |
| All | 117/354565 | 33(23.7-35.2) | 187/569644 | 33(28.4-38.1) | 35/120150 | 29(19.4-41.64) | 42/201770 | 21(15.1-28.4) |
| Age-adjusted to Brazil | | 29.5(20.7-30.8) |  | 33.2(28.6-38.3) |  | 29.4(20.24-41.65) |  | 27.5(19.8-37.2) |
| Age-adjusted to World | | 33.1(23.5-46.3) |  | 40.7(29.4-54.5) |  | 34.7(24.4-47.5) |  | 30.6(21.1-42.8) |

TOAST: Trial of ORG 10172 in Acute Stroke Treatment; CI: confidence interval; IS: ischemic stroke.

**Supplementary figure 1 -** Incidence of ischemic stroke by TOAST classification


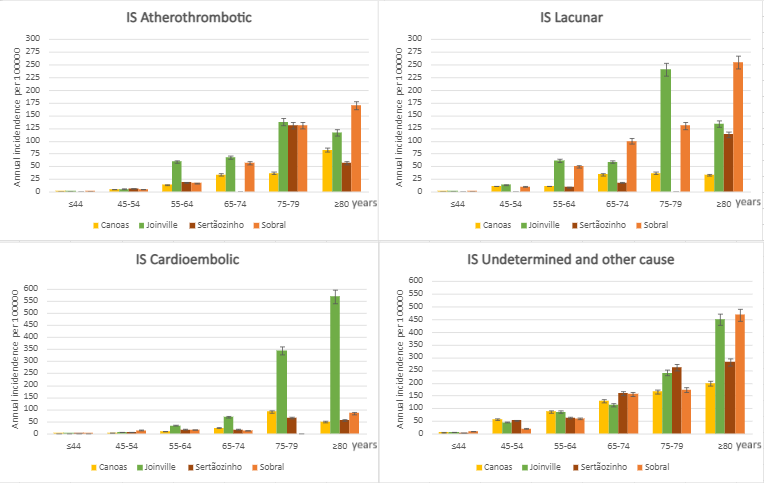


IS: ischemic stroke
